# Explaining organisational responses to a board-level quality improvement intervention: findings from an evaluation in six providers in the English National Health Service

**DOI:** 10.1136/bmjqs-2018-008291

**Published:** 2018-10-31

**Authors:** Lorelei Jones, Linda Pomeroy, Glenn Robert, Susan Burnett, Janet E Anderson, Stephen Morris, Estela Capelas Barbosa, Naomi J Fulop

**Affiliations:** 1 School of Health Sciences, Bangor University, Bangor, UK; 2 Department of Applied Health Research, University College London, London, UK; 3 Florence Nightingale Faculty of Nursing, Midwifery and Palliative Care, King's College London, London, UK; 4 Centre for Patient Safety and Service Quality, Imperial College London, London, UK

**Keywords:** governance, quality improvement, organizational theory

## Abstract

**Background:**

Healthcare systems worldwide are concerned with strengthening board-level governance of quality. We applied Lozeau, Langley and Denis’ typology (transformation, customisation, loose coupling and corruption) to describe and explain the organisational response to an improvement intervention in six hospital boards in England.

**Methods:**

We conducted fieldwork over a 30-month period as part of an evaluation in six healthcare provider organisations in England. Our data comprised board member interviews (n=54), board meeting observations (24 hours) and relevant documents.

**Results:**

Two organisations transformed their processes in a way that was consistent with the objectives of the intervention, and one customised the intervention with positive effects. In two further organisations, the intervention was only loosely coupled with organisational processes, and participation in the intervention stopped when it competed with other initiatives. In the final case, the intervention was corrupted to reinforce existing organisational processes (a focus on external regulatory requirements). The organisational response was contingent on the availability of ‘slack’—expressed by participants as the ‘space to think’ and ‘someone to do the doing’—and the presence of a functioning board.

**Conclusions:**

Underperforming organisations, under pressure to improve, have little time or resources to devote to organisation-wide quality improvement initiatives. Our research highlights the need for policy-makers and regulators to extend their focus beyond the choice of intervention, to consider how the chosen intervention will be implemented in public sector hospitals, how this will vary between contexts and with what effects. We provide useful information on the necessary conditions for a board-level quality improvement intervention to have positive effects.

## Introduction

Worldwide, healthcare systems are concerned with strengthening board-level governance of quality.^1–3^ In England, national healthcare regulators are developing approaches, resources and interventions aimed at supporting senior hospital leaders in their role in the governance of quality.^4 5^ There is increasing recognition of the value of organisational theory for understanding the implementation of quality improvement (QI) interventions.^6 7^ In this study, we apply organisational theory^8^ to describe and explain the response of healthcare provider organisations to a board-level QI intervention aimed at supporting senior hospital leaders to develop an organisation-wide QI strategy. We adopt a contingent approach^9^ concerned with identifying the critical variables that influence an organisation’s response to a QI intervention.

### Organisational responses to QI interventions

We are not aware of any previous studies of board-level interventions in the public healthcare sector. Looking at the effectiveness of improvement interventions more generally, a number of studies have explored how the cultural, structural and political characteristics of the adopting organisation shape its response. These contextual factors may create difficulties in embedding and sustaining changes to practices, and produce unintended consequences.^8 10–14^

In their study of the introduction and impact of management interventions in publicly funded healthcare organisations in Canada, Lozeau, Langley and Denis^8^ argue that as interventions introduced into public sector hospitals are often borrowed from the private sector, there is a ‘compatibility gap’ between the assumptions underlying the design of the intervention and the characteristics of the adopting organisation. For example, the adoption of ‘strategic planning’ by an organisation assumes that senior leaders have the autonomy to make and implement decisions. This assumption may be incompatible with public sector hospitals, where leadership is fragmented, power diffuse and where there are multiple goals (eg, patient care and cost control). The compatibility gap produces a mismatch between the intended and actual use of the intervention. From their findings, Lozeau *et al* developed a typology of possible organisational responses (table 1).

**Table 1:** Organisational responses to a quality improvement intervention^8^

Response	
Transformation	The organisation modifies its processes with the result that the intervention has the intended effect.
Customisation	The organisation adapts the intervention in the process of changing organisational processes.
Loose coupling	The intervention is implemented in a superficial way with organisational processes remaining largely unaffected.
Corruption	The intervention becomes captured and distorted to reinforce existing organisational roles and power structures.

Research in the National Health Service (NHS) in England^10 13^ found that in addition to ‘bottom-up’ corruption from professionals within the organisation, interventions can also undergo ‘top-down’ corruption, driven by the demands of central government. When the intended objectives of the intervention were to promote education, collaboration and knowledge sharing, these were displaced by the quantifiable outcomes prioritised by central government. This research also found an erosion of staff engagement with the intervention over time.^10^ This was not a case of staff ‘resistance’, as there was no evidence of resistance to the improvement goals per se, rather it involved

an unwillingness (or, rather, inability) to be actively involved in improvement work unless supported by the provision of protected time and recruitment of additional clinical members of staff, neither of which is possible without substantial financial investment. (p. 1178).

The compatibility gap highlighted by studies of NHS organisations in England relates to the differences between relatively autonomous private sector organisations and public sector organisations that operate in a target-driven, policy-dependent and resource-constrained context.

We contribute to this literature by analysing the responses of six hospital boards to an improvement intervention. In contrast to previous research, outlined above, this intervention was developed specifically for public sector hospitals, based on research on mainly publicly funded hospitals.^15 16^ We addressed the following questions: (1) How do public sector hospital boards respond to a QI intervention, and what are the effects? (2) How do features of the wider social and political context shape the response? (3) What are the implications for the future design and implementation of interventions aimed at enhancing the capability of boards to improve the quality of patient care?

### Intervention

The intervention facilitated the use of a research-based guide for senior hospital leaders to develop and implement organisation-wide QI strategies.^15^ The guide is based on findings from the QUASER study of quality in hospitals, a collaboration between five European countries (England, Norway, Sweden, Netherlands, Portugal) funded by the EU FP7 programme.^17^ The use of the guide was facilitated in six provider organisations by Foresight Partnership (an organisational development consultancy), now part of GE Healthcare, between July 2014 and May 2015. The intervention involved a self-assessment questionnaire, which was completed by both executive and non-executive members of the board (10–12 in each organisation). Each organisation nominated three board members to attend a series of learning events. In addition to developing an organisation-wide QI strategy, participants were given the task of implementing one organisation-wide QI project. More information on the intervention is provided in the supplementary material.

10.1136/bmjqs-2018-008291.supp1 Supplementary data

## Methods

We report findings from a qualitative study, undertaken between April 2014 and December 2016, as part of an evaluation of the intervention. Qualitative methods are appropriate for our research questions which are concerned with understanding how, and why, the intervention worked (or not) to distill lessons that can be applied to future interventions.^18^ We used a combination of methods (interviews with participants, observations of board meetings and document analysis) to triangulate our findings and provide an in-depth contextualised understanding of the process of organisational improvement.

### Sample

Nine organisations in an urban/suburban area were invited to participate in the intervention as part of a research study, of which six agreed. The fee for participating for each organisation was £5000 (paid to Foresight as a contribution to the cost of providing the intervention). Boards were offered an alternative programme that provided a more bespoke and intensive form of consultancy at a higher cost, but all opted for the lower cost option. The profiles of participating organisations are given in table 2.

**Table 2:** Profiles of participating organisations

	1	2	3	4	5	6
Type	Acute	Mental health	Acute	Acute	Acute	Acute
Foundation trust*	Yes	Yes	No	Yes	No	Yes
Performance (as rated by the national regulator)	Outstanding	Requires improvement	Requires improvement	Good	Requires improvement	Good

*In England, the majority of healthcare providers are now ‘foundation trusts’ (142/242, 62%). Foundation trusts are, in principle, granted greater autonomy over finances and service development than non-foundation trust providers.

### Data collection

Data were collected across three phases—‘before’, ‘during’ and ‘after’ the intervention (table 3). At each time period, we observed board meetings, taking notes on what was discussed, by whom and for how long, together with descriptions of interactions and our interpretation of board dynamics. We also interviewed members of the board. We asked boards to nominate members to be interviewed, both executive and non-executive, including the non-executive director responsible for quality. Interviews, which were audio-recorded and transcribed, were semistructured on the topic of governance of QI. Interviews conducted during and after the intervention asked participants about their views and experiences of the intervention and their progress with the tasks (developing an organisation-wide QI strategy and development and implementation of one organisation-wide QI project). We collected a range of documents for each organisation (board meeting papers, annual reports, Quality Accounts, project reports and PowerPoint presentations) for additional information on the extent to which organisations had developed an organisation-wide QI strategy and the nature of QI activities.

**Table 3:** Summary of data collection

Period	Methods
Before (T1): April 2014 to July 2014	Interviews with between 4 and 6 members of each participating board (n=30) Observation (1 meeting of each board) Document analysis of papers from the meeting we observed and an additional set of minutes; Quality Accounts (a mandatory report on the quality of services provided by an organisation and published annually); rolling 5-year strategy; quality committee minutes; inspection reports from national regulators
During (T2): July 2014 to April 2015	Observation (workshop and action learning sets) Interviews with 1–2 participants from each board (n=9)
After (T3): May 2015 to December 2016	Repeat interviews with board members, where these individuals were still in post and willing to participate (n=24), and any additional staff members leading on the development and implementation of the nominated project (n=2) Observation (1 meeting of each board) Document analysis of meeting papers and other relevant documents, as in T1. We also requested documents—reports, PowerPoint presentations etc—relating to the implementation of the nominated project

### Analysis

During an initial phase of data familiarisation, LJ suggested that Lozeau, Langley and Denis’ model would be a helpful conceptual framework for analysis. Having established an empirical fit with this model, subsequent analysis focused on describing and explaining the response of the organisations in our study to the intervention, and explicating the contingencies which shaped this response. Data were organised in a table, using the procedures for framework analysis.^19^ LJ then drafted a narrative for each case, as recommended for studies of organisational processes.^20^ These were interpretive accounts developed from the raw data (interview accounts, board observations and documents), describing the response of each organisation to the intervention and suggesting analytical linkages between the organisational response and contextual features. The table and narratives were circulated and discussed among the research team.

## Findings

Responses to the intervention varied across the organisations in our study. Two organisations transformed work processes in response to the intervention. These organisations responded to the intervention as intended, producing an organisation-wide QI strategy, and made substantial progress implementing one organisation-wide QI project. One organisation customised the intervention, with a positive consequence in the form of accomplishing locally identified priorities for QI. In two of the remaining organisations, the response could be characterised as loose coupling with no positive effects attributable to the intervention. In the final organisation, the intervention was corrupted by external regulatory demands (see online supplementary table S2 for detail). We found that the response of organisations to the intervention was contingent on two main contextual features, the availability of ‘slack’ and the functioning of the board. These features are elaborated below. Data extracts have been chosen to illustrate our findings.

### Slack

In our study, ‘slack’ comprised positive conditions for both thought and action. These two components were frequently referred to by participants in our study as having ‘thinking space’ and ‘someone to do the doing’. The amount of slack that was available to organisations was shaped by the extent to which organisations were compliant with national standards, such as the Department of Health’s standard for a maximum of 4 hours patient waiting time in accident and emergency (A&E) departments. Where organisations had not met external standards, activities were focused on meeting these, and the organisation faced increased pressure from external bodies, such as commissioners and regulators. This reduced their autonomy and placed additional demands and requirements on the organisation, such as additional reporting, and attendance at meetings with external organisations. The autonomy gained from meeting national standards also facilitated the acquisition of funds for QI. For example, organisation 1 was able to negotiate with the commissioner dedicated funding for QI initiatives. This provided funding for additional staff to ‘do the doing’.

Where organisations lacked thinking space or capacity for action, the response to the intervention diverged from what was intended. For example, organisation 3 was not compliant with a number of national standards and in the year prior to the intervention it had been placed in ‘special measures’ by the national regulator. This is a process imposed on organisations when the overall service, or certain core services, have been rated ‘inadequate’. The organisation is required to follow an externally imposed ‘improvement plan’ and is subject to a follow-up review to assess performance against the plan. This organisation also faced severe financial constraints and lacked key resources. For example, as a result of a previous cost cutting exercise, all clinical governance posts had been removed. Although initial engagement with the intervention was strong, the key stumbling block was finding someone to ‘do the doing’, especially in relation to developing and implementing an organisation-wide QI project.

The intervention competed with the improvement plan imposed by the regulator which was clearly the top priority for the organisation. This was explicit in the interview accounts following the intervention:

So what I am trying to say is basically in the scheme of things if this was helping me get out of special measures the answer would be absolutely I would be there. At the moment it’s not helping me get out of special measures. (Chair, T3)

Following the intervention, the organisation was allocated, by central government, to an alternative organisational development initiative. Thus, despite the intervention having apparent traction with senior leaders, and strong initial engagement, it was dislodged by competing priorities and eventually displaced by another initiative imposed by central government.

Organisation 5 was also struggling to meet a number of national standards. As a non-foundation trust, the organisation was subject to performance management from the NHS hierarchy and additional reporting requirements from a range of external agencies. In this case, the intervention was subsumed into externally driven imperatives for quality assurance and the application for foundation trust status (see table 2). Analysis of documents revealed that the intervention was used primarily to prepare the required documents for the foundation trust application. Implementation of an organisation-wide QI project stalled when the organisation failed to meet the A&E access targets. At the same time, the national regulator inspected the A&E department and rated it inadequate, requiring the organisation to submit an improvement plan to the regulator.

In the case of organisation 6, the intervention was one of a large number of quality and safety initiatives that the organisation was participating in. The intervention was seen as ‘just another initiative’, separate to other QI projects, and attended to in a superficial way. Over time, commitment to the intervention was diluted as it competed with other initiatives for participants’ time and attention, reflected in declining attendance at the learning events (see online supplementary table S1). When it came to implementing an organisation-wide QI project, a number of existing initiatives were ‘re-labelled’^21^ as the nominated project for the intervention. There was no evidence that the intervention modified organisational processes.

### Functioning of the board

The organisations that benefited from the intervention had stable leadership and a shared vision for QI. In contrast, organisation 3 had a history of organisational turbulence and lacked stable leadership due to continuous turnover of the executive and non-executive teams. Staff who took up executive roles on the board were often referred to as ‘holding the fort’ and ‘stepping up’. This suggests an important ‘compatibility gap’, between the assumption, in a board-level QI intervention, that there is a coherent and functioning governing entity, and the reality, which in the case of organisation 3 was a fluid collection of individuals with temporary responsibilities. This gap, between the board as imagined in the intervention, and the board in practice, was referred to directly by the chair during an interview:

What you have here is a bit different from the normal board, you have got very experienced non exec directors, not so experienced executive directors. So therefore the board is not like the board written about in a Foresight kind of document, it’s very much like seasoned operators with people who have stepped up to take a very tricky job. (Chair, T3)

Organisation 5 had poor relationships between board members, especially between the medical director and the director of nursing, and between the medical director and the director of operations. In the year following the intervention, key members of the board, including the medical director, the CEO and the chair, left the organisation. At 12-month follow-up, none of the members of the board who had participated in the intervention remained at the organisation.

Organisation 6 also lacked a functioning board. One executive director claimed that decision-making happened entirely at the level of the Executive Board, with the Board of Directors serving only to ratify the decisions of the executive team. Both interview accounts and board observations suggested that the Board of Directors played no effective role in debating decisions, developing strategy, or in scrutinising and challenging the performance of the organisation. One interviewee suggested that the board was a performance, noting that ‘everything at the board is highly rehearsed’ (medical director, T1). Interview accounts suggested that the response to the intervention reflected a broader organisational strategy of superficially or ritualistically attending to governance of QI, as the director of nursing observed, ‘I think we play at it’ (T1).

## Discussion

We found that the organisational response to a board-level QI intervention was contingent on the availability of slack and the functioning of the board. Slack has previously been found to play an important role in improving organisational performance, enabling organisations to initiate strategy and proactively adopt innovations.^22^ In relation to QI, slack has been defined as the ‘cushion of resources within an organisation that facilitates innovation and change by providing crucial time and support for learning and creativity to occur’ (p. 102).^23^ Research on hospitals that have sustained QI has stressed that ‘slack is not a ‘surplus’ or a ‘luxury’ but something that has to be built into an organisation for it to continually support innovation and improvement’ (p. 107). The implications of our research for regulators concerned with strengthening board-level governance of quality is that they need to take slack seriously and reduce accordingly the number of demands on organisations. Organisations considering engaging in this type of initiative might first consider what they could ‘stop doing’ to enable sufficient slack. Our study suggests that simply adding one more initiative to an already overburdened organisation will not produce the desired results.

Organisations considering engaging in a board-level QI initiative also need to ensure that there is in fact a board. We found that there was not always a coherent and functioning governing body. In organisation 3, for example, there was what we have termed a ‘nominal board’. In this organisation, a very high turnover of board membership resulted in available staff ‘stepping up’ to fill a board-level role in the absence of alternative candidates, resulting in a board ‘in name only’. In organisation 6, there was what we have termed a ‘staged board’. In this organisation, the board of directors was a performance of decisions that had already been made by the executive directors. While governance practices often have a performative dimension,^24^ we use the category ‘staged board’ to denote a governing body characterised by a cynical focus on display, and control of information, and where insufficient trust and openness inhibits challenge, debate and effective action.

Recent research has highlighted the extent of ‘churn’ in board membership in England.^25^ A study of 145 provider organisations found that vacancies in the executive team were widespread, and the median tenure of an executive director was 3 years. In low-performing organisations (as rated by the national regulator), 14% of posts were vacant and 72% of executives had been appointed in the previous year. In comparison, in high-performing organisations, only 3% of posts were vacant and 20% of executives had been appointed within the past year. The study found that the problem is exacerbated by a tendency, on the part of some national bodies and politicians, to ‘personalise’ poor performance, making it harder to recruit individuals to these roles.

The consequences of churn include a focus on day-to-day priorities at the expense of longer-term strategy. High turnover can also stall organisational and service development, and short tenures undermine the credibility executive directors have with staff in their own organisations and with external stakeholders. There is, therefore, an important role for regional and national bodies in supporting senior leaders and developing their capabilities.^25^ However, while these measures may help to address the problem of the ‘nominal board’, remedying a ‘staged’ board is likely to be more challenging. In our case, the functioning of the board was prevented by the unproductive use of power by long-standing individuals. Our findings can inform the design of board-level interventions, which should be adapted to the context of individual organisations, for example, by focusing on developing the building blocks of healthy board functioning in organisations where this is weak.

Our findings support those of previous empirical studies in showing how a QI intervention can be corrupted by the demands of the centre (in this case the Department of Health and other national bodies which set systems for regulation and performance management). Our findings indicate an incompatibility between an intervention that aims to foster learning and develop long-term strategy for QI, and the short term-focus required by the procedures put in place by national bodies for assurance.

In the case of organisation 3, participation in the intervention was only loosely coupled with organisational dynamics; however, this did not involve, as previous studies describe it, a superficial or ritualistic participation in the intervention. Rather, individuals were prevented from taking any action due to an absence of necessary slack. We suggest that, in the context of public sector hospitals, loose coupling can be understood as inaction or ‘stalling’, as well as the more familiar superficial or ritualistic action. This inaction can also be induced by the centre, as when the Department of Health intervened to allocate organisation 3 to a new, national initiative.

A limitation of our study was that the majority of data collection was at board level. This ‘top-down’ focus neglects the influence of other features of the organisational context, such as the professional workforce.^8^

## Conclusion

Underperforming organisations, under pressure to improve, have little time or resources to devote to organisation-wide initiatives such as those described here, highlighting a paradox in that the organisations most in need of such interventions are unlikely to benefit from them. Our research highlights the need for policy-makers and regulators to extend their focus beyond the choice of intervention, to consider how the chosen intervention will be implemented in public sector hospitals, how this will vary between contexts and with what effects. We provide useful information on the necessary conditions for a board-level QI intervention to have positive effects.
