# Supplementary material for: Explaining organisational responses to a board-level quality improvement intervention: findings from an evaluation in six providers in the English National Health Service
Source: BMJ Qual Saf. 2018 Oct 31;28(3):198–204. doi: 10.1136/bmjqs-2018-008291 (PMC6560458; doi:10.1136/bmjqs-2018-008291)
Supplement: Supplementary data [file bmjqs-2018-008291supp001.pdf]

**Supplementary material**

**The iQUASER intervention**

The intervention involved the following stages:

*1. Individual organisational self-assessment using the QUASER Guide*. Foresight Partnership (FP) created an on-line self-assessment survey based on the guide. This survey took approximately 30 minutes to complete. FP recommended that this was completed by the following individuals: All executive directors, including the chief executive, chair, non-executive director with lead for quality, all other non-executive directors who wanted to take part, and, at the discretion of the board, divisional directors (clinical leaders) and/or those with a senior QI lead role not on the board. From the results of the self-completed assessment tool FP generated a report for each organisation to discuss and build a shared view of where the key quality improvement challenges are for each organisation. On the basis of the results of the self-assessment questionnaire each organisation was asked to:

i. Identify the areas of quality improvement that they feel need particular attention in their organisation

ii. Select one whole-organisation quality improvement intervention to pursue over the year of this programme

iii. Nominate three organisational leaders to participate in an initial workshop and 'action learning set'[1], and two follow-up action learning sets at 4 and 8 months. It was recommended that this included one executive director and one clinical leader. It was also

suggested that participants may also include the non-executive director who chairs the organisation's quality-focused subcommittee.

*2. QUASER Workshop and Action Learning Set.* This was a one-day workshop attended by senior leaders from all the participating organisations. The focus for the morning session was on the research that underpins the QUASER guide[2,3] and on developing a collective understanding of the QUASER guide as a framework for developing elements of an organisation's quality improvement strategy. The afternoon session was the first of three action learning sets. Two executive members of each participating organisation were allocated to small groups. The intention was that membership of these groups would remain constant throughout the programme.

The aim of the first action learning set was to create a supportive environment and for participating organisations to draw on the collective experience and wisdom of all the participants so as to:

i. Further develop an understanding of what approaches will be most effective to build an organisation-wide quality improvement strategy, based on each organisation's current stage of development, and addressing the areas of quality improvement that may need most attention from their self-assessment.

ii. To explore and develop approaches to implementing an organisation-wide quality improvement intervention.

By the end of the day it was anticipated that all participants would have arrived at specific goals or commitments to take their quality improvement strategies and interventions forward.

*3. Follow Up Action Learning Sets.* Two follow-up facilitated action learning sets (at 3 and 6 months) explored implementation challenges, and ways to overcome them, as well as following up on progress. In addition the final learning set asked participants to reflect on the value of the guide in facilitating QI, as well as the value of facilitated support for implementation. Attendance at the learning events is given in table S1.

**Table S1. Attendance at the iQUASER intervention (workshop and action learning sets)**

| Org. | Workshop and ALS 1 | ALS 2 | ALS 3 |
|---|---|---|---|
| 1 | Chief Executive<br>Director of Organisation and Transformation<br>Non-executive director | Chief executive<br>Director of Organisation and Transformation | Chief Executive<br>Director of Organisation and Transformation |
| 2 | Chair<br>Director of Nursing<br>Chief Operating Officer<br>Non-executive director (Quality Lead) | Director of Nursing<br>Chief Operating Officer | Chief Operating Officer |
| 3 | Chair<br>Medical Director<br>Chief Nurse | Medical Director<br>Director of Planning and Governance | Director of Planning and Governance |
| 4 | Director of Nursing<br>Clinical lead (consultant)<br>Non-executive director<br>Non-executive director<br>Non-executive director | Medical Director<br>Clinical lead | Medical Director<br>Clinical lead |
| 5 | Director of Nursing<br>Clinical lead (nurse)<br>Medical Director<br>Non-executive director<br>Associate Director of Governance | Medical Director<br>Associate Director of Governance | Associate Director of Governance |
| 6 | Medical Director<br>Director of Quality and Safety<br>Non-executive director (quality lead) | Medical Director<br>Divisional Medical Director (quality) | Did not attend |

**Table S2. The organisational response to the quality improvement intervention and subsequent effects**

| Organisation | 1 | 2 | 3 | 4 | 5 | 6 |
|---|---|---|---|---|---|---|
| Response | Transformation | Customisation | Loose coupling | Transformation | Corruption | Loose coupling |
| Slack | Met all external standards<br>Recently appointed a director of organisation and transformation<br>Negotiated a dedicated funding stream for QI from the commissioner. This funding was used to employ an organisation-wide QI team. | Not fully compliant with external standards | Received a poor performance rating from the healthcare regulator and subject to additional scrutiny and assurance activities.<br>Previous disinvestment in clinical governance staff to save money. | Met all external standards<br>Appointed a director of quality who was responsible for the activities associated with the intervention. | Competing priorities, particularly meeting external standards and applying for Foundation Trust status.<br>Greater regulatory burden due to status as a non-foundation trust.<br>During the course of the study the organisation received a poor performance rating from the healthcare regulator and subject to additional scrutiny and assurance activities. | A large number of QI initiatives. |

| | | | | | | |
|---|---|---|---|---|---|---|
| Functioning of the board | Stable leadership<br>The board had already spent some time reflecting on QI.<br>Shared recognition of the need to take a systematic approach. | Currently focused on quality assurance but recognition of the need to focus more on strategy.<br>During the period of the study there was a change in CEO and medical director. | Organisational turbulence and unstable leadership. High turnover of executive director team. Interim medical director.<br>No shared vision or strategy. | Stable leadership<br>Shared vision | New CEO<br>Difficult relationships on the board between members of the executive team, especially between the medical director and the director of nursing, and between the medical director and the director of operations.<br>Short-term focus<br>At 12 month follow-up no original members of the executive team were still in post. | Board a highly rehearsed 'performance'.<br>No shared understanding, or strategic direction, of QI at board-level.<br>Short-term focus |
| Engagement<br>With the intervention | Strong | Moderate | Minimal | Strong | Minimal | Moderate |
| QI strategy | Improved | Improved | Not improved | Improved | Not improved | Improved |
| Implementation of an organisation-wide QI project | Large extent | Large extent | Not at all | Large extent | Not at all | Moderate extent |

| | | | | | | |
|---|---|---|---|---|---|---|
| QI project | An organization-wide QI facility to coordinate QI activities across the organization. | (1) Review of governance arrangements.<br>(2) Organisation-wide no smoking policy<br>(3) Improve bed management (eliminate external placements and non-clinical bed transfers). | None selected | Diabetes project | None selected | Pre-existing initiatives 're-labelled' as the QI project |
| Outcome | Developed an organisation-wide QI strategy<br><br>Appointed a director of QI as part of an organisation-wide QI facility. | Used intervention to think about and accomplish local objectives | Displaced by another QI intervention allocated by central government | Developed an organisation-wide QI strategy<br><br>Appointed a director of QI responsible for coordinating QI across the organisation. | Focus on external demands for quality assurance<br><br>Intervention used to meet requirements for application to foundation trust status. | Seen as separate to other QI initiatives and attended to in a superficial way.<br><br>Nothing new or different as a result of the intervention. |

1. Marquardt M. Building the learning organisation. New York: McGraw-Hill 1996.

2. Burnett S, Mendel P, Nunes F, Wiig S, van Bovenkamp H, Karltun A, Robert G, Anderson J, Vincent C, Fulop NJ. Using Institutional Theory to Analyse Hospital Responses to External Demands for Finance and Quality in Five European Countries. J Health Services Research & Policy 2016; 21 (2): 109-117. doi:10.1177/1355819615622655.

3. Wiig S, Aase K, von Plessen C, Burnett S, Nunes F, Weggelaar M, Anderson-Gare B, Calltorp J, Fulop N. Talking about quality: exploring how 'quality' is conceptualized in European hospitals and healthcare systems. BMC Health Services Research 2014, 14:478. DOI: 10.1186/1472-6963-14-478
